# EGFR induces DNA decomposition via phosphodiester bond cleavage

**DOI:** 10.1038/srep43698

**Published:** 2017-03-08

**Authors:** Yongpeng Tong, Shuiming Li, Chunliu Huang

**Affiliations:** 1 College of Physics and Energy, Shenzhen University, Shenzhen, 518060, China; 2 College of Life Science and Oceanography, Shenzhen University, Shenzhen, 518060, China; 3 Zhongshan School of Medicine, Sun Yat-Sen University, Guangzhou, 510080, China

## Abstract

EGFR may induce DNA degradation. This activity had not been previously described as an EGRF function. To confirm this unexpected activity, testing of EGFR in the presence of ATP and either 5A, 5C, 5G, 5T, or 5U oligonucleotides was performed. HPLC-MS analysis demonstrated that 5A and 5U levels significantly decreased in the presence of EGFR. Furthermore, fragments 4A and 4U were produced in 5A+EGFR+ATP and in 5U+EGFR+ATP reaction mixtures, respectively, but not in EGFR-negative controls. Degradation of Poly(A), Poly(C), Poly(G), Poly(I), Poly(T), and Poly(U) oligomers in the presence of EGFR and ATP correlated with the lower ability of reaction products to pair with complementary oligonucleotides. Gel electrophoresis showed that breakdown products migrated more quickly than controls, especially after addition of paired (complementary) oligomers, Poly(A) and Poly(U). Furthermore, λ DNA reaction products also migrated more quickly after incubation with EGFR. The results suggest that EGFR can induce breakage of certain types of nucleotide phosphodiester bonds, especially within the A residues of DNA or U residues of RNA, to induce DNA or RNA decomposition, respectively. This activity may be important in EGRF signaling, DNA degradation, or repair in normal or cancer cell activities.

Previous studies have shown that cellular signaling mediated by the epidermal growth factor receptor (EGFR) plays a key role in numerous signaling pathways. One such example is the control of proliferation and differentiation of cortical progenitor cells (CPCs), where regulatory mechanisms involved in EGFR signaling remain largely unknown. It has been reported that necdin, a MAGE (melanoma-associated antigen) family protein may interact with EGFR in primary CPCs to repress downstream EGFR signaling linked to astrocyte differentiation. Indeed, the generation of autophosphorylated EGFR with subsequent necdin interaction has been observed in EGF-stimulated CPCs^1^. In addition, the growth factors EGF and bFGF (also known as FGF-2) are indispensable for maintaining self-renewal and multipotency of neural stem cells residing in the developing mammalian telencephalon^2^. Furthermore, in the early stages of embryonic cortical development, CPCs proliferate and differentiate into neuronal progenitors in response to bFGF; in the late stages they differentiate into glial progenitors in response to EGF. These results align with the fact that the EGF receptor (EGFR, also known as ErbB1 or HER1), a member of the receptor tyrosine kinase family, is expressed in the embryonic cortex at low expression levels during early development and at high levels during the late period^3^. Finally, mutant mice lacking the EGFR gene exhibit abnormal development and postnatal cerebral cortical neurodegeneration^4^, indicating that EGFR plays an important role during normal cortical development.

A multifunctional role of EGFR has been postulated, due to observations of several interesting characteristics regarding this receptor. First, the extracellular domain of EGFR can adopt two conformations, either closed or extended, whereby the latter form is dimerisation-competent. Following binding to its cognate ligand(s), EGFR forms dimers capable of autophosphorylation and of phosphorylating other proteins. EGF binding stabilises the extended form, thus favouring dimer formation, which allows the EGFR kinase domain to access its Tyr substrates. After EGFR dimerisation, the tyrosine kinase domain of one EGFR moiety phosphorylates several Tyr residues in its partner moiety. This kinase activity is countered by phosphatase activity acting at the membrane during the very early stages of EGFR signaling^5^. Notably, abundant studies also indicate that EGFR activation is a causal driver of many cancers, including breast, lung, brain, and colourectal cancers. Furthermore, activating mutations observed in KRAS and BRAF, which are essential downstream effectors of EGFR, are among the most common mutations found in a very wide range of human cancers^6^. Many questions still remain unanswered before we can assess the importance of EGFR and its downstream effectors in normal cell function and in carcinogenesis.

EGFR also participates in EGFR/Ras/MAPK signaling. However, the precise linkage between EGFR/Ras/MAPK signaling and cell growth and division is surprisingly obscure for animal cells in general. While it is known that EGFR promotes intestinal stem cell proliferation, the mechanism underlying this observation is still poorly understood. Notably, one potentially important downstream target of EGFR signaling is the HMG-box transcriptional repressor, Capicua (Cic). This highly conserved DNA binding factor has been shown to act downstream of receptor tyrosine kinase (RTK)/Ras/MAPK signaling in *Drosophila* eye and wing imaginal discs, embryos, and ovaries. There it regulates diverse RTK-dependent processes including cell proliferation, specification, and pattern formation. Cic orthologues in both invertebrate and vertebrate species share two well-conserved regions: the HMG-box, presumed to mediate DNA binding at target promoters, and a C-terminal domain^7^,^8^.

Other clues regarding EGFR functions have been uncovered due to the fact that EGFR is a tyrosine kinase that has often been used to study the ATP-dependent phosphorylation of protein Tyr residues. Tyrosine kinase (TK) signaling has garnered much interest in recent years, principally in cancer research, due to its demonstrable success as a point of action for precision targeting of drugs that act upon critical pathogenic drivers^9^. Under regulated conditions, tyrosine phosphorylation acts as a rapid on-off switch in cells and is employed by cellular signaling pathways to regulate growth, migration, adhesion, differentiation, and survival, as well as proliferation signals to promote tumor development. Moreover, tyrosine kinases are activated in cells upon DNA damage and, in turn, activate signal transduction networks required to restore cellular homeostasis^10^,^11^.

Notably, multiple studies have demonstrated that EGF can also stimulate tumor growth via several mechanisms: phosphorylation of EGFR tyrosine residues, ultimate activation of ERK (extracellular signal-regulated kinase) pathways, and possible up-regulation of proliferative genes after intranuclear localisation of phosphorylated EGFR. Furthermore, EGF treatment has been observed to act downstream of up-regulated genes via phosphorylation, protein modification, amino acid phosphorylation, and phosphorus metabolism^12^. Although direct EGFR-induced breakdown of nucleotides has not been previously reported, it is well known that EGFR’s tyrosine kinase activity could induce ATP phosphodiester bond breakdown via a substrate phosphorylation process. Therefore, it is possible that RNA or DNA base residues, which possess features in common with ATP structure, may also react with EGFR to bring about RNA or DNA decomposition via an analogous phosphodiester bond breakage process. Moreover, EGFR-induced DNA breakdown at specific sequence positions has been observed to up-regulate gene expression and even induce further gene mutation accumulation and subsequent cancer development^13^. Furthermore, over-expression of EGFR activates the phosphoinositide 3-kinase pathway, including survival (Akt/pAkt) and proliferation (Erk/pErk) factors that activate DNA-dependent protein kinase (DNA-PK), leading to repair of irradiation-damaged DNA^14^,^15^. Due to the numerous studies above that ascribe a DNA degradative function to EGFR, this study was conducted to determine if EGFR directly induces breakdown of special nucleotides in DNA, an activity that may possess special biological significance.

## Results and Discussion

**Figure 1:**
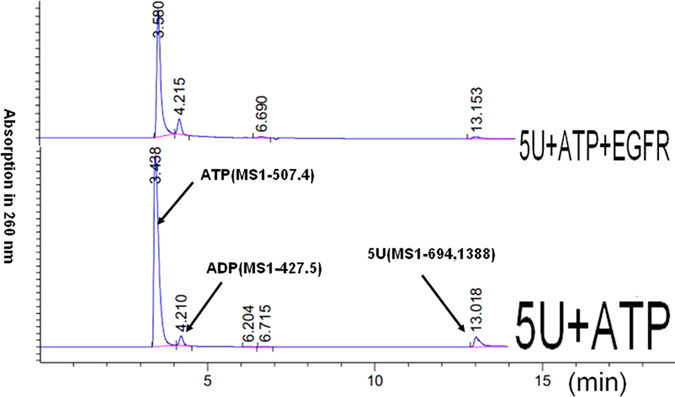
A typical HPLC-MS spectrum (absorbance measured at 260 nm) comparing reaction of the deoxyribonucleotide oligomer 5U in the presence of ATP with or without human EGFR/HER1/ErbB1 (aa668–1210).

From Fig. 1 and Table 1, the relative concentrations of 5A, 5G and 5U were significantly decreased after reaction with EGFR (as compared to the ATP content, which is in excess and assumed constant during the reaction). Compared to those controls without EGFR, 5A, 5G and 5U levels in the presence of EGFR were significantly decreased.

**Figure 2:**
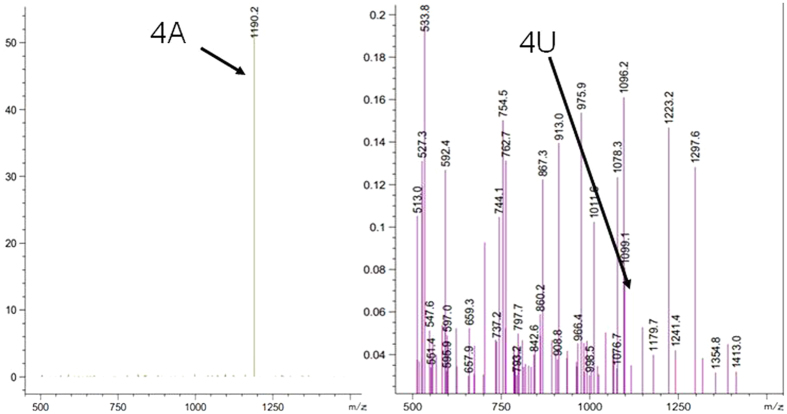
A typical MS of the 4A (MS2–1190.2) fragment in 5A+EGFR+ATP solution and the 4U (MS2–1099.1) fragment in the 5U+EGFR+ATP reaction mixture.

Figure 2 displays results obtained after testing of five kinds of deoxyribonucleotides (5A, 5C, 5G, 5T, 5U) for degradation in the presence of human EGFR/HER1/ErbB1 and ATP. It is clear that the 4A and 4U fragments (the breakdown products of 5A and 5U, respectively) are observed, but 4C, 4G and 4T fragments are not observed. Therefore, only 5A and 5U may have decomposed in the presence of EGFR. The per cent breakdown of phosphodiester bonds of 5A resulting in conversion to 4A was approximately 0.5% and for 5U conversion to 4U was less than 0.1%, as calculated using the peak area ratio (4A/5A or 4U/5U). No detectable decomposition was observed in the control incubated without EGFR and ATP (fragment peaks using LC/MS cannot be detected).

**Figure 3:**
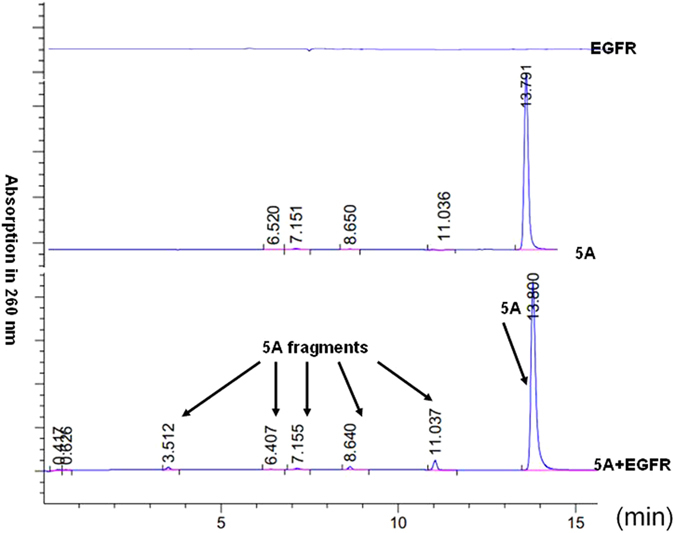
A typical HPLC-MS spectrum (absorbance measured at 260 nm) comparing reaction of the deoxyribonucleotide oligomer 5A with or without human EGFR/HER1/ErbB1 (aa668–1210).

From Fig. 3, the quantify percentage of the breakdown of phospholipid bonds of 5A can be analyzed by HPLC. After a quantified calculation of the total 5A fragment peak area (subtract the background from the controls)/5A peak area, the percentage of breakdown parts of 5A in 5A+EGFR solution at this experimental condition is about 4.6%.

At first, ATP is thought to be helpful for the breakdown reaction as it is necessary for EGFR autophosphorylation, ultimately, we found that the breakdown reaction did not need ATP. The mechmism may be that adenine nucleotide (A) residues in DNA are competed with ATP to combine the “ATP pocket” position of EGFR and then the DNA breakdown reaction is happened.

**Figure 4:**
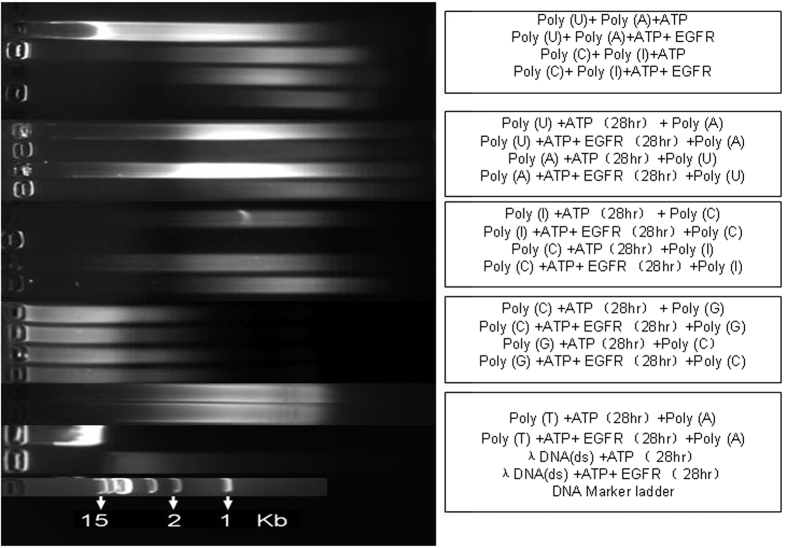
Alkaline agarose gel electrophoresis of ssPoly(X) paired products after incubation in the presence of ATP either with or without EGFR are shown. Lanes (1–4) show reaction products after 37 °C incubation for 28 h for: Poly(U)+Poly(A)+ATP, Poly(U)+Poly(A)+ATP+EGFR; Poly(C)+Poly(I)+ATP, Poly(C)+Poly(I)+ATP+EGFR, respectively. Lanes (5–8) show reaction products for: (Poly(U)+ATP (37 °C, 28 h))+Poly(A) 0.5 h, 19 °C), (Poly(U)+ATP+EGFR (37 °C, 28 h))+Poly(A) 0.5 h, 19 °C), (Poly(A)+ATP (37 °C, 28 h))+(Poly(U) 0.5 h, 19 °C), (Poly(A)+ATP+EGFR (37 °C, 28 h))+Poly(U) 0.5 h, 19 °C), respectively. Lanes (9–12) show products of the reactions: (Poly(I)+ATP (37 °C, 28 h))+Poly(C) 0.5 h, 19 °C), (Poly(I)+ATP+EGFR (37 °C, 28 h))+Poly(C) 0.5 h 19 °C), (Poly(C)+ATP (37 °C, 28 h))+Poly(I) 0.5 h, 19 °C), (Poly(C)+ATP+EGFR) (37 °C, 28 h))+Poly(I) 0.5 h, 19 °C), respectively. Lanes (13–16) show products for: (Poly(C)+ATP (37 °C, 28 h))+Poly(G) 0.5 h, 19 °C), (Poly (C)+ATP+EGFR (37 °C, 28 h))+Poly(G) 0.5 h, 19 °C), (Poly(G)+ATP (37 °C, 28 h))+Poly(C) 0.5 h, 19 °C), (Poly(G)+ATP+EGFR (37 °C 28 h))+Poly(C) 0.5 h, 19 °C), respectively. Lanes (17–21) show reaction products for: (Poly(T)+ATP (37 °C, 28 h))+Poly(A) 0.5 h, 19 °C), (Poly(T)+ATP+EGFR (37 °C, 28 h))+Poly(A) 0.5 h, 19 °C), λ DNA(ds)+ATP (37 °C, 28 h), λ DNA(ds)+ATP+EGFR (37 °C, 28 h), and DNA marker ladder with fragment mixtures (1, 2, 3, 5, 8, 15 kbp), respectively.

Figure 4 shows results of alkaline agarose gel electrophoresis. Alternate pairs of lanes display results for reactions without or with EGFR during the first incubation; even-numbered lanes contain EGFR, while odd-numbered lanes do not.

Lanes (1–2) show that the paired DNA in the mixture (Poly(U)+Poly(A)+ATP) (lane 1) exhibited a higher fluorescence intensity and a lower electrophoretic mobility than for an identical formulation incubated in the presence of EGFR (lane 2). A similar result was also observed for the mixture (Poly(C)+Poly(I)+ATP) (lanes 3–4).

Lanes (5–18) also show the effect of EGFR on reaction products. The mixture solution was incubated in two steps. In step 1, one Poly(X) without EGFR (odd-numbered lanes) or Poly(X) with EGFR (even-numbered lanes) was incubated with ATP for 28 h at 37 °C first and then in step 2, a second complementary strand Poly(X) was added to the corresponding mixture solution for further incubated at 19 °C for 0.5 h. Results in lanes 5–16 exhibit similar patterns in that each reaction without EGFR exhibited a higher fluorescence intensity and a lower electrophoretic mobility than the same mixture with EGFR. Thus, analogous effects of EGFR were observed for (Poly(U)+ATP+Poly(A) (lanes 5–6), (Poly(A)+ATP+Poly(U) (lanes 7–8), (Poly(I)+ATP+Poly(C) (lanes 9–10), and Poly(C)+ATP+Poly(I) (lanes 11–12). However, (Poly(C)+ATP+Poly(G) (lanes 13–14), (Poly(G)+ATP+Poly(C) (lanes 15–16), and (Poly(T)+ATP+Poly(A) (lanes 17–18), show that the presence of EGFR did not affect subsequent fluorescence intensity or electrophoretic mobility of the paired DNA.

Lanes (19–20) show that after incubation for 28 h at 37 °C, the products of λ DNA+ATP solution exhibited both higher fluorescence intensity and lower electrophoretic mobility without EGFR than after incubation with EGFR. The result suggests that EGFR may react with DNA bases by inducing base dephosphorylation and DNA degradation.

Lane 21 shows the DNA size marker (1 kb-III, GENEray).

It is well known that staining of double-stranded DNA (dsDNA) or double-stranded RNA (dsRNA) using ethidium bromide or other intercalating dye results in much higher fluorescence intensity than stained single-stranded DNA or RNA^16^. Furthermore, dsDNA and dsRNA also exhibit lower electrophoretic mobility than their denatured single-stranded counterparts, as visualised using alkaline agarose gel electrophoresis. From the above experimental results, it can be deduced that ssDNA (Poly(A), Poly(U), and Poly(I)) may decompose or become phosphorylated by EGFR, as their products exhibited a lower fluorescence intensity and a higher electrophoretic mobility than controls lacking EGFR. Moreover, dsDNA may also decompose or become phosphorylated upon reaction with EGFR, as dsDNA reaction products also exhibited a lower fluorescence intensity and a higher electrophoretic mobility than controls. Notably, significant conversion of ssDNA (Poly(C), Poly(G) and Poly(T)) was not observed after incubation with EGFR.

**Figure 5:**
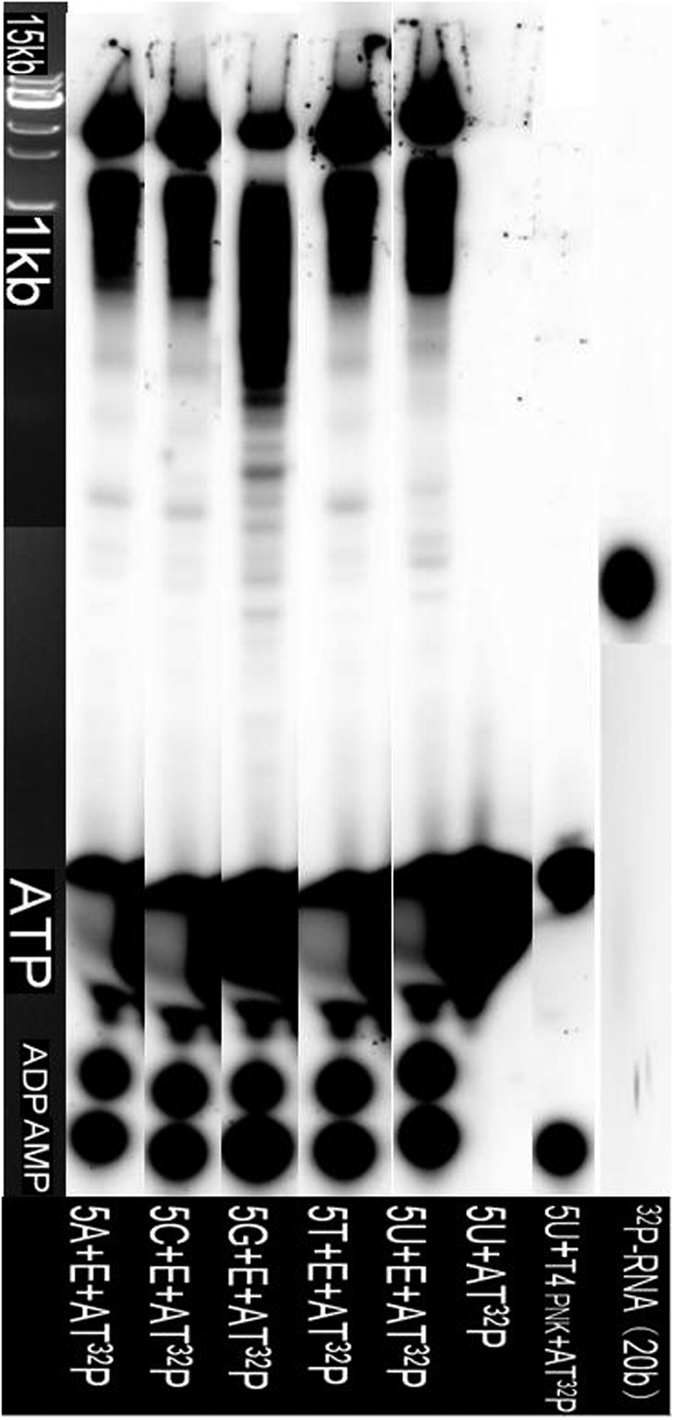
The ^32^P-ATP autoradiography study of the phosphorylation of 5X (A, C, G, T, U) by EGFR.

From Fig. 5, 5X phosphorylation products were not produced, as no ^32^P-labelled bands were observed in the 2b–20b size range on PAGE gels. Only EGFR-^32^P self-phosphorylated products and ^32^P-AMP, ^32^P-ADP, ^32^P-ATP were observed. The result suggests that EGFR/HER1/ErbB1 can only induce breakdown of phosphodiester bonds of some nucleotides, especially residues containing A or U, causing DNA or RNA instability and decomposition.

As a final note, by reconciling the results reported in this study with structural data regarding the EGFR catalytic site, we can present a possible scheme to explain the mechanism behind EGFR degradation of DNA. After the first structure of an ATP+Mg^2+^-dependent protein kinase was solved, HER1/EGFR was studied for the next 20 years as a potential therapeutic target for the treatment of various human cancers. It was subsequently found that the principal EGFR catalytic site was an ATP-binding pocket. This site is the target of many physiological regulators and most experimental or therapeutic inhibitors, which typically block its activity in a competitive or allosteric fashion. In this study, the ATP-binding pocket may also be an important site where EGFR can excise an A or U residue of DNA or RNA, causing DNA or RNA instability and decomposition.

Although the breakdown reaction does not need ATP, the breakdown reaction experiments of DNA and EGFR in the presence of ATP is also important as ATP exists commonly in the living cells.

**Figure 6:**
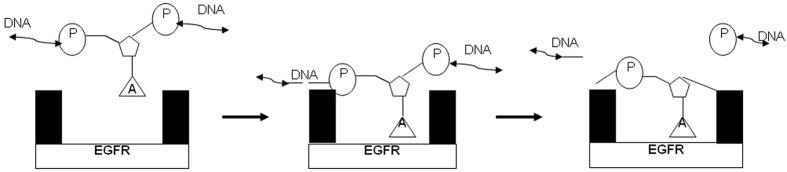
The scheme of DNA breakdown induced by EGFR.

The suggested scheme is shown in Fig. 6.

Figure 6 scheme – a novel EGFR binding mechanism with RNA or DNA might be exploited for optimisation of the design of an EGFR inhibitor for future cancer treatment.

From the above scheme, in most cases EGFR can induce single-stranded DNA or RNA breaks in certain special positions, as there is low probability of adenine nucleotides (A) lying adjacent to one another in double-stranded DNA. Furthermore, breakdown of double-stranded RNA or DNA by EGFR may be also related to the adjacent molecular environment (surrounding sequence or the presence of other proteins). Therefore, EGFR-induced single-stranded DNA or RNA breakdown may participate in up-regulation of gene expression normally induced during cellular events, such as repair of Okazaki fragments during normal DNA synthesis.

## Conclusion

All of the above experiments uniquely illustrate that EGFR, a tyrosine kinase, can induce breakage of certain phosphodiester bonds, especially in A or U residues within DNA or RNA, to induce changes in gene expression that may promote the development of cancer. However, a deeper understanding of the mechanics involved in the maintenance of genome stability as part of a larger EGFR (EGFR different isoforms and mutants) regulation scheme is needed.

## Methods

In order to demonstrate a direct effect of EGFR on RNA or DNA integrity, several pilot experiments were first conducted to optimise reaction conditions (incubation time, ATP concentration, and enzyme concentration). Using agarose gel electrophoresis, DNA breakdown was most clearly observed using the initial reagent concentrations as follows: EGFR (0.02–0.10 μg/μl), λ DNA (0.15 μg/μl), and ATP (25 mM), with an incubation time of at least 28 h.

Using reagent concentrations optimised in the pilot study, five deoxynucleotide oligomers were tested as EGFR-induced breakdown targets. The oligomers, 5A, 5C, 5G, 5T, and 5U, were synthesised by Sangon Biotech Co., Ltd. (Shanghai, China). Of a 1.0 mM stock solution for each oligomer, 50 μl was used in a reaction with 4 μl of a 0.52 μg/μl stock solution of human EGFR/HER1/ErbB1 (aa668–1210). The recombinant human EGFR/GST chimera, consisting of 780 amino acids with a calculated molecular mass of 89.1 kDa, was purchased from Sino Biological Inc. (PA, USA) (>85% purity). ATP was added (50 μl of a 50 mM stock solution) (Sangon Biotech Co., Ltd, >98% purity) and reaction mixtures were adjusted to a final 1X T4 PNK reaction buffer A concentration using 10X stock (Thermo Scientific, NY, USA). Reaction mixtures containing reaction buffer without EGFR and ATP were used as controls.

After incubation for 28 h at 37 °C, reactions were filtered using Pall Microsep centrifugal devices (10 K, 1.5 ml) and 5 μl of each filtrate was analyzed using LC/MS. An Agilent 6120 LC/MS system (a triple quadrupole mass spectrometer) was used to analyze solutions containing 5X+ATP with or without EGFR. An Agilent Single Quadrupole LC/MS (G6120, Agilent, CA, USA) with multimode source for positive and negative ionisation (ESI+and ESI− mode) and Agilent MassHunter Walkup Software for LC/MS and LC systems were used for data acquisition. UV detection was performed at 260 nm. The analytes were separated using an Ecosil C18 column (250 mm × 4.6 mm; Guangzhou Lubex Biological Technology Co., Ltd., China). The mobile phase consisted of a mixture of (A) 0.1% formic acid (LC/MS grade) and (B) methanol (LC/MS grade). A gradient elution method was used (Time:0-2-16-20-25-25.1-30 min using solvent A:0%-0%-40%-90%-90%-0%-0%). Milli-Q water was used in all experiments.

A typical HPLC spectrum is shown in Fig. 1 and the HPLC analysis of 5X (X = A, C, G, T, U) relative content (compared to ATP) after reaction with or without EGFR (absorption at 260 nm) is shown in Table 1. A corresponding typical fragment MS result is shown in Fig. 2.

After incubation of EGFR (0.04 μg/μl), 5A (0.5 mmol) and 5A(0.5 mmol)+EGFR(0.04 μg/μl) solution respectively for 28 h at 37 °C, the quantify percentage of the breakdown of phospholipid bonds of 5A can be analyzed by HPLC. The corresponding result is shown in Fig. 3.

Agarose gel electrophoresis of the paired ssPoly(X) experiment was also performed after Poly(X) (X = A, C, G, I, T, and U) reacted with or without EGFR in the presence of ATP. 10 μl (0.4 mg/ml) Poly(X) (purity >90%) (Sangon Biotech Co., Ltd.) was used to react with or without 4 μl 0.52 μg/μl EGFR with 10 μl 50 mM ATP reaction solution and reactions were adjusted to 1X T4 PNK reaction buffer A using 10X stock. After incubation for 28 h at 37 °C, 10 μl (0.4 mg/ml) corresponding paired (complementary) single-stranded DNA-Poly(X) was added and cultured for 0.5 h at 19 °C. Also, 5 μl 0.3 μg/μl λ DNA (*E. coli* W3350 dsDNA, 48.5 kb, Sangon Biotech Co., Ltd.) was separately tested with or without 4 μl 0.52 μg/ml EGFR with 10 μl of 50 mM ATP reaction buffer at 37 °C for 28 h. After incubation, 10 μl of each reaction mixture was analysed using agarose gel electrophoresis (each 1% gel containing 20 g of agarose in 200 ml volume was stained using #41003 GelRed DNA stain (Biotium Inc., CA, USA). Each gel was 6 cm in length. Electrophoresis was performed using 1X TAE running buffer with settings of 80 V, 50 mA and a running time of 35 min at room temperature. The result is shown in Fig. 4.

In order to determine if EGFR induces RNA and DNA decomposition or phosphorylation, a ^32^P-ATP autoradiographic study was conducted using polyacrylamide gel electrophoresis (PAGE). Reactions containing 4 μl of 20 μM deoxynucleotide-oligomers 5X (5A, 5C, 5G, 5T, 5U) were tested for degradation after addition of 1 μl human EGFR/HER1/ErbB1 (aa668–1210), 2 μl [γ-^32^P]-ATP (3000 Ci/mmol, 10 mCi/ml, PerkinElmer Co., CA, USA) and 3 μl reaction buffer solution (10X T4 PNK reaction buffer A using 10X stock). After incubation for 28 h at 37 °C, 5 μl of each reaction was electrophoresed on denaturing 7 M urea PAGE gels (15 cm × 15 cm) using 1X TBE running buffer and 10 W of power for 2 h at room temperature. After separation, the gel was covered with a phosphor screen to collect photo-stimulated luminescence from the imaging plate after exposure to γ radiation from the ^32^P-gel for 3 h. Next, the phosphor screen was read using a Typhoon FLA 7000 laser scanner (GE Healthcare, UK). Lane 1 shows the DNA ladder (1kb-III, GENEray, PRK) used for a size marker (detected by luminescent detection). Lanes (2–6) show the scanning map of the 5X+EGFR+^32^P-ATP reaction mixture and lanes (7–9) show the scanning map of the control (^32^P-RNA, 20b) marker obtained by reaction of RNA (20b) with T4 PNK in the presence of ATP). The result is shown in Fig. 5.

## Additional Information

**How to cite this article:** Tong, Y. *et al*. EGFR induces DNA decomposition via phosphodiester bond cleavage. *Sci. Rep.* **7**, 43698; doi: 10.1038/srep43698 (2017).

**Publisher's note:** Springer Nature remains neutral with regard to jurisdictional claims in published maps and institutional affiliations.

## Figures and Tables

**Table 1:** HPLC analysis of 5X (A, C, G, T, U) relative content (compare to ATP) after react with or without EGFR (absorption in 260 nm).

	5A*	5C	5G*	5T	5U*
A_260(5X)_/A_260(ATP)without_	0.16 ± 0.04(5)	0.12 ± 0.06(5)	0.20 ± 0.06(5)	0.03 ± 0.02(5)	0.073 ± 0.03(5)
A_260(5X)_/A_260(ATP)with_	0.12 ± 0.03(5)	0.13 ± 0.06(5)	0.11 ± 0.04(5)	0.02 ± 0.03(5)	0.023 ± 0.02(5)

^*^Significant differences by T-test for two groups comparison.
